# Wireless Motion Variability Analysis with Integrated Triboelectric Textiles via Displacement Current

**DOI:** 10.1021/acsnano.4c18766

**Published:** 2025-05-23

**Authors:** Yinghong Wu, Sunil Kumar Sailapu, Chiara Spasiano, Carlo Menon

**Affiliations:** † Biomedical and Mobile Health Technology Group, Department of Health Sciences and Technology, 27219 ETH Zürich, Lengghalde 5, Zürich 8008, Switzerland; ‡ National Engineering Research Center of Green Recycling for Strategic Metal Resources, Institute of Process Engineering, Chinese Academy of Sciences, Beijing 100190, China; § School of Industrial and Information Engineering, Politecnico di Milano, Milan 20133, Italy

**Keywords:** wearable technology, triboelectric textile, displacement current, wireless physiological monitoring, motion variability

## Abstract

Triboelectric textiles have recently garnered significant attention for their ability to detect and analyze body movements. However, the transmission of wireless signals from natural human motion via integrated triboelectric wearables has been hampered by the reliance on nontextile components, physical interface spacers, and wired connections. Here, we report a wireless biosensing triboelectric wearable system that exploits inherent microgaps within electrospun nanofibers to create a triboelectric textile, seamlessly woven into the garment at various positions. This design enables untethered continuous biosignal monitoring, including those indicative of stride-time variability and joint-time variability analysis from daily activities and exercise. Signals are wirelessly transmitted via displacement current—enhanced by a textile inductor—to an external reader. This innovation enables wireless signal transmission directly from garments without the need for external energy sources. This work advances the development of fully textile-based wireless wearable sensors contributing to next-generation motion tracking, health monitoring, and smart clothing technologies.

## Graphical abstract

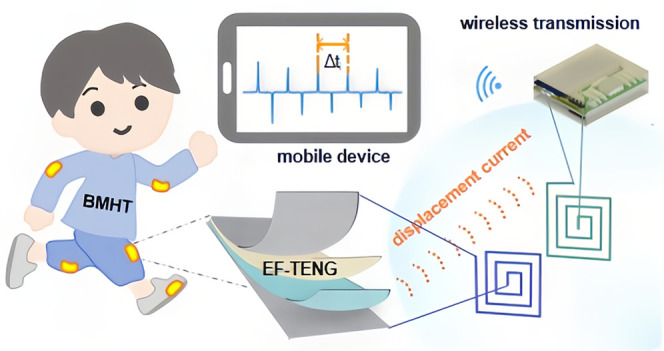

## 1 Introduction

Human movement of varying speeds and intensities shapes health, rehabilitation, and athletic performance. Capturing movement variability across different body locations—such as stride time variability (STV) for gait analysis and joint time variability (JTV) for upper-body motion—provides deeper insights into mobility, coordination, and neuromuscular control. STV, defined as fluctuations in step timing, serves as an important indicator of balance and fall risk, while JTV reflects joint motion consistency, which is relevant for motor function assessment. Wearable devices like fitness trackers and smartwatches monitor body movement beyond clinical settings.^1–3^ Smart textiles, integrating electronics in fabrics, can push the boundaries by transmitting activity data wirelessly.^4,5^ Among these, the textile-based triboelectric nanogenerator (t-TENG), which generates electricity based on contact electrification and electrostatic induction, has emerged as a cutting-edge motion-sensing technology.^6–8^ However, the application of t-TENGs for comprehensive movement variability analysis remains underexplored, primarily due to the challenges associated with device configuration and signal transmission.

From the device configuration perspective, the seamless integration of t-TENGs into garments is imperative for not only the accurate capture of movement signals for variability analysis but also for ensuring unobtrusive and comfortable wear for everyday use. However, the majority of previously reported t-TENGs^9–11^ failed to be integrated into clothing, primarily due to the artificial physical spacer at the interface. Consequently, the development of spacer-free t-TENGs with microgaps is desirable to achieve a balance between effective contact-separation operation for enhanced output and device integration for comfortable wearability. In this regard, numerous efforts have been made to develop core–shell fibers,^12,13^ single-electrode textiles,^14,15^ and woven or knitting fabrics.^16,17^ These designs, however, often either require external triboelectric components or complicate device configuration, burdening the manufacturing process and hindering the scalability of production. In contrast, electrospun nanofibers, which employ a more straightforward and scalable approach,^18^ hold considerable promise for the fabrication of spacer-free, full-textile triboelectric wearables, as demonstrated by prior studies.^19,20^ By eliminating the need for physical spacers while creating inherent microgaps, these nanofibers can be more closely integrated with clothing, offering a seamless and less obtrusive user experience. Therefore, we hypothesize that an electrospun full-textile TENG (EF-TENG) is a promising candidate for variability analysis.

Traditional wireless transmitters require an external power source for continuous operation, posing challenges for fully self-sustained wireless transmission in wearable-sensing applications. From the signal transmission perspective, traditional wireless approaches—even those adapted for TENGs in different applications—are inadequate for real-time motion tracking and further variability analysis due to several limitations. *First*, TENGs lack the capacity to directly power commercial wireless transmitters. They require intermittent charging of storage components such as capacitors or batteries to operate the transmitters.^21,22^ This adds circuit complexity and hinders real-time data transmission. In the context of analyzing human motion, these data gaps between charging states can impact the derivation of metrics such as gait cycles and STV. *Second*, battery-powered wireless transmitters,^23–25^ though capable of real-time communication of TENG signals, introduce uncomfortable bulk on garments and negatively impact user comfort due to physical connectors and maintenance requirements. *Third*, some wireless strategies, utilizing capacitors and inductors for resonant transmission, necessitate manual or electronic switching,^26,27^ increasing design complexity and relying on nontextile elements. Recent advancements enable wireless signal transmission through dynamic electric fields generated by TENG alone,^28–30^ eliminating external power supplies or commercial wireless transmitters. This technique offers a simplified readout by directly capturing the characteristic TENG signal at operating frequency, bypassing the need for fast sampling or large data volumes typical of resonant approaches,^31,32^ making it promising for extended activity monitoring.

This work explores an enhanced displacement–current-sensing approach for wireless movement monitoring and variability analysis using a microgap EF-TENG wearable, investigating its feasibility for activity tracking. Our design eliminates the need for physical spacers commonly used between triboelectric layers, allowing seamless integration into daily clothing and real-time wireless sensing. Notably, the EF-TENG demonstrated sustained performance and functionality, even after extended operation and long periods of storage. A key advancement is improved wireless communication, achieved by incorporating a textile inductor alongside the EF-TENG to significantly boost the transmitted signal strength. By wirelessly capturing the transmitted dynamic electric fields—further enhanced with a textile inductor—using a custom-developed reader, our fully textile biosensing system physically isolates the reader from the wearable. While the external reader requires power for signal acquisition, the EF-TENG and the inductor enable self-sustained wireless transmission without an external power source on clothing. This not only eliminates the need for wired connectors, rigid electronics, and external power sources on clothing but also enables continuous, untethered activity tracking for variability analysis over extended periods.

## 2 Results and Discussion

A standard EF-TENG comprises four layers: two conductive fabric electrodes separated by two electrospun textile layers—poly­(vinyl chloride) and nylon (Figure 1a). It is noteworthy that the focus of this study is not on the selection of a specific triboelectric pair but rather on the natural motion tracking and variability analysis using a fully textile TENG within a wireless communication framework. The choice of triboelectric materials and device design was guided by our previous study,^33^ which identified two key selection criteria. First are materials with high electron affinity differences, which increase triboelectric charge densities at the interface and further the output, especially for cases without physical spacers. Second are materials with nontoxic, waterproof, and biocompatible properties, which benefit the device’s wearability and durability. Additionally, we systematically evaluated different conductive fabric electrodes and found that while silver-coated fabric provides higher conductivity, nickel-coated fabric offers superior flexibility and mechanical durability when integrated into clothing (Figure S1). As the electrode choice had a minimal impact on device output but significantly affected comfort and long-term wearability, we selected nickel-coated fabric for all wearable applications in this study. Importantly, the microgaps between electrospun nanofibers (Figure S2) not only make sure the contact-separation cycle occurs without the need for physical spacers but also facilitate the seamless integration of the device and its subtle motion tracking.

**Figure 1:**
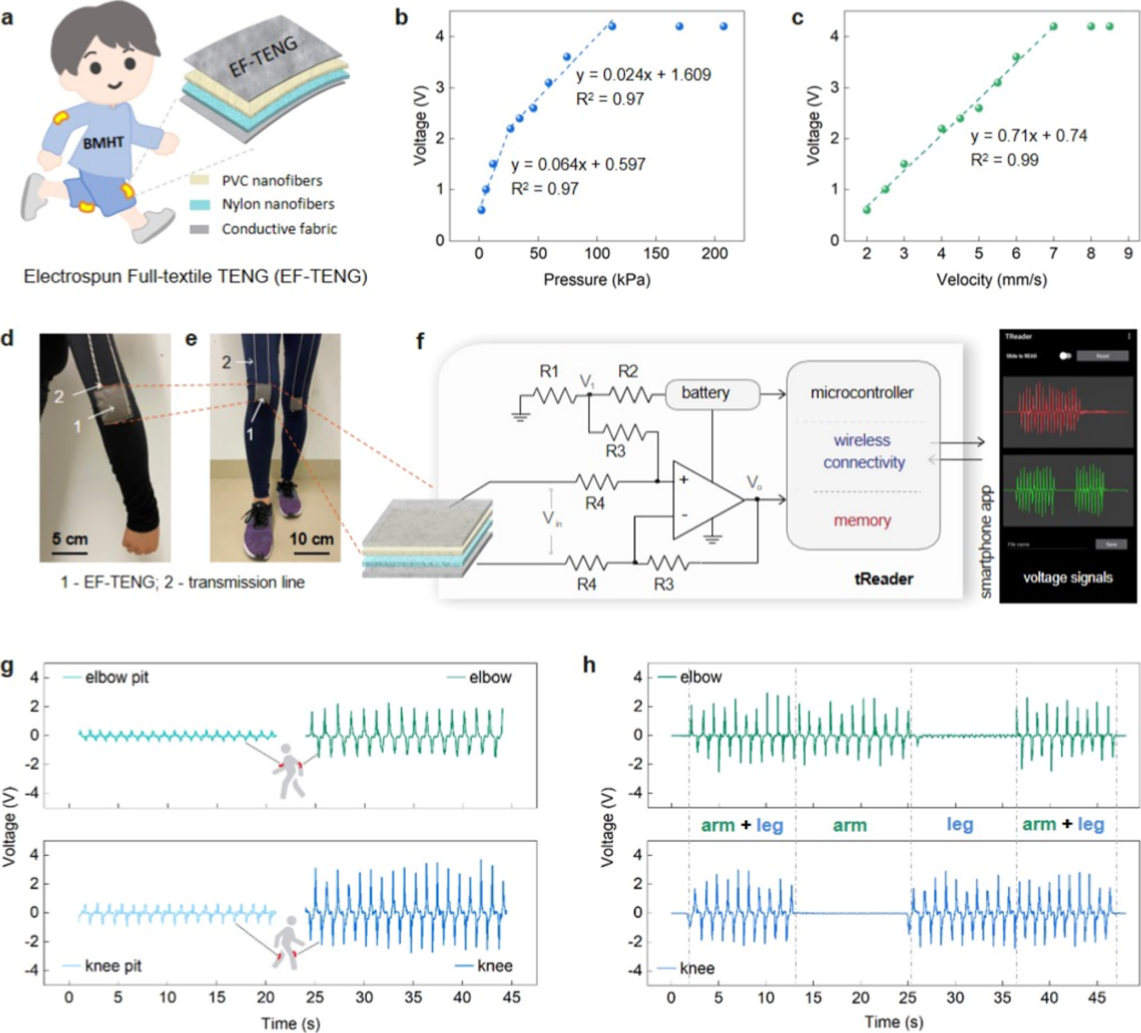
Integration of EF-TENG and its wearable applications in human motion tracking via tReader. (a) Schematic diagram of a typical EF-TENG and its integration into everyday clothing. (b) Voltage characteristics of EF-TENG for different pressures. (c) Voltage characteristics of EF-TENG for loading velocities. (d,e) Photographs of the EF-TENGs integrated on the garment and worn near the elbow and knee areas. (f) Scheme illustrating blocks of tReader connecting to the smart garment and communicating response wirelessly to a smartphone app. The picture shows a screenshot of the smartphone app with output voltage signals. (g) Comparison of voltage signals generated from EF-TENG at different positions on the smart garment. (h) Voltage generated during simultaneous motion tracking of arm and leg by voluntarily controlling their movements while walking.

To investigate the feasibility of integrating EF-TENG into everyday clothing for motion tracking, its sensitivity was assessed to different pressures and loading velocities by individually controlling each parameter. The voltage–pressure characteristics obtained by systematically recording the output response within a range of 2–200 kPa (Figure 1b) revealed two linear regions in the ranges of 0–30 and 30–110 kPa, with no significant change over 110 kPa. The sensitivities observed in these regions are 64 mV/kPa and 24 mV/kPa, respectively. The relatively lower sensitivities can be attributed to a trade-off between maximizing signal stability and resolution over the amplitude (see Section 4.5), spacer-free constraints imposed by the device design to ensure durability and seamless textile integration, and gentler testing conditions designed to simulate human motion. The loading velocity at the same frequency also significantly affected the response of the EF-TENG (Figure 1c). The output voltage increased linearly with the loading velocity between 2 and 7 mm/s, with a sensitivity of 710 mV s/mm. This velocity range corresponds to realistic displacements in wearable applications, considering the micro- to millimeter-level proximity between the EF-TENG and the motion source during natural human movement. As the frequency of human motion is relatively random and diverse, the frequency of the generated voltage signals was also compared to loading frequencies between 0.5 and 5 Hz, which encompasses the motion associated with typical daily human activities. The frequency of generated signals with EF-TENG was identical to loading frequencies, confirming its ability to respond to rapid body movements (Figure S3).

On this basis, our microgap-featured EF-TENGs were sewn on the everyday clothing and worn around the elbow and the knee regions of the garment, as shown in Figure 1d,e. To assess the sensitivity of EF-TENG to human daily motions, the initial tests were performed using hand and leg movements to simulate low-impact exercises. However, instead of any wire-connected equipment such as an electrometer or oscilloscope, to avoid limitations of restricted and unnatural assessment during long-term activity tracking, we developed a lightweight pocket reader. This reader enables continuous signal acquisition from EF-TENG by connecting directly to the transmission lines via detachable connectors. It acquires averaged but quality-improved data points at a suitable interval (in this case, 15 ms) for decoding motion-related parameters. The design of the reader features a circuit, as illustrated in Figure 1f, that centrally incorporates an operational amplifier to handle the generated signal levels from EF-TENG and feed them to the microcontroller in a suitable range. The output voltage *V*_o_ of the operational amplifier can be expressed by the following equation:

1 $$V_{o}=V_{1}+V_{in}\frac{R_{3}}{R_{4}}$$

The circuit scales the voltage generated by the EF-TENG using resistors *R*_3_ and *R*_4_ and introduces an offset using *V*_1_ to accommodate the voltage level in a suitable range for the analog-to-digital converter on the microcontroller. The microcontroller converts the fed signal to report the generated voltage by accounting for the scaling and offset values set by the circuit. The battery-powered tReader wirelessly communicates this information in real-time to a custom-designed smartphone application.

By residing in the garment’s pocket, the reader enabled unrestricted user movement while capturing the smart garment’s rapid responses to body movements. The EF-TENG produced concurrent voltage peaks for arm and leg bending and release. The measurements indicated the output signals with EF-TENGs on the elbow and knee (chosen for further studies) to be 4–5 times higher than those obtained with those on the elbow pit and knee pit (Figure 1g). These results indicate the possibility of conveniently tailoring EF-TENG to different clothing locations and modulating the signal nature based on the desired application. We also investigated the smart garment’s response to motion on an inclined plane while ascending and descending stairs (Figure S4). The generated electrical signals in this case are of a similar nature to those observed earlier. More importantly, the designed independent device provided us with the opportunity to demonstrate the ability of the reader to simultaneously track different body movements during normal walking by voluntarily controlling arm and leg movements (Figure 1h, Video S1). The high sensitivity and rapid response of our EF-TENGs make it feasible to achieve sensing at multiple locations simultaneously, which holds promise in aiding patients in detecting the coordination of their body movements during rehabilitation. The signal with the EF-TENG is found reproducible when tested with four separate EF-TENGs for normal walking, with a typical average peak value of ∼ 3.0 ± 0.2 V (Figure S5). The EF-TENGs also maintained consistent performance over a 10 month period of storage, delivering an average peak value of ∼3 V (Figure S6), indicating long-term stability suitable for real-world use. While different materials, fabrication approaches, and circuit design could enhance signal strength, we prioritized achieving a single voltage peak with a well-defined amplitude for each bending and release of the arm or leg. This distinct peak is crucial for deriving useful motion-related metrics during further extensive continuous monitoring studies.

Real-time monitoring of body movement in naturalistic settings holds immense potential for improving the diagnosis and treatment of gait and posture disorders, helping to mitigate the risk of falls, neurodegenerative disorders, and multiple sclerosis. The EF-TENG delivers a single voltage peak for each leg movement, and the interval between peaks helps extract motion-related metrics like the number of gait cycles, stride time, and its variability. The EF-TENG and tReader systems were evaluated for continuous physiological monitoring by deriving these metrics while running on a treadmill with stepwise increasing speeds from walking to running. A gyroscope was also placed on the runner’s shoe to derive similar metrics (Figure 2a). The average stride time (Δ*t*) was measured by averaging the intervals between immediate signal peaks (Δ*t*_*i*_) in the obtained readings (Figure 2b). The Δ*t* and its variability (STV) obtained with the EF-TENG and gyroscope at different speeds agreed closely with each other with a maximum difference of 5 ms and 0.16%, respectively (Figure 2c). The gait cycle number was also calculated by counting the signal peaks within a fixed duration for different speeds. The EF-TENG and gyroscope indicated the same gait cycle number, as shown in Figure 2d.

**Figure 2:**
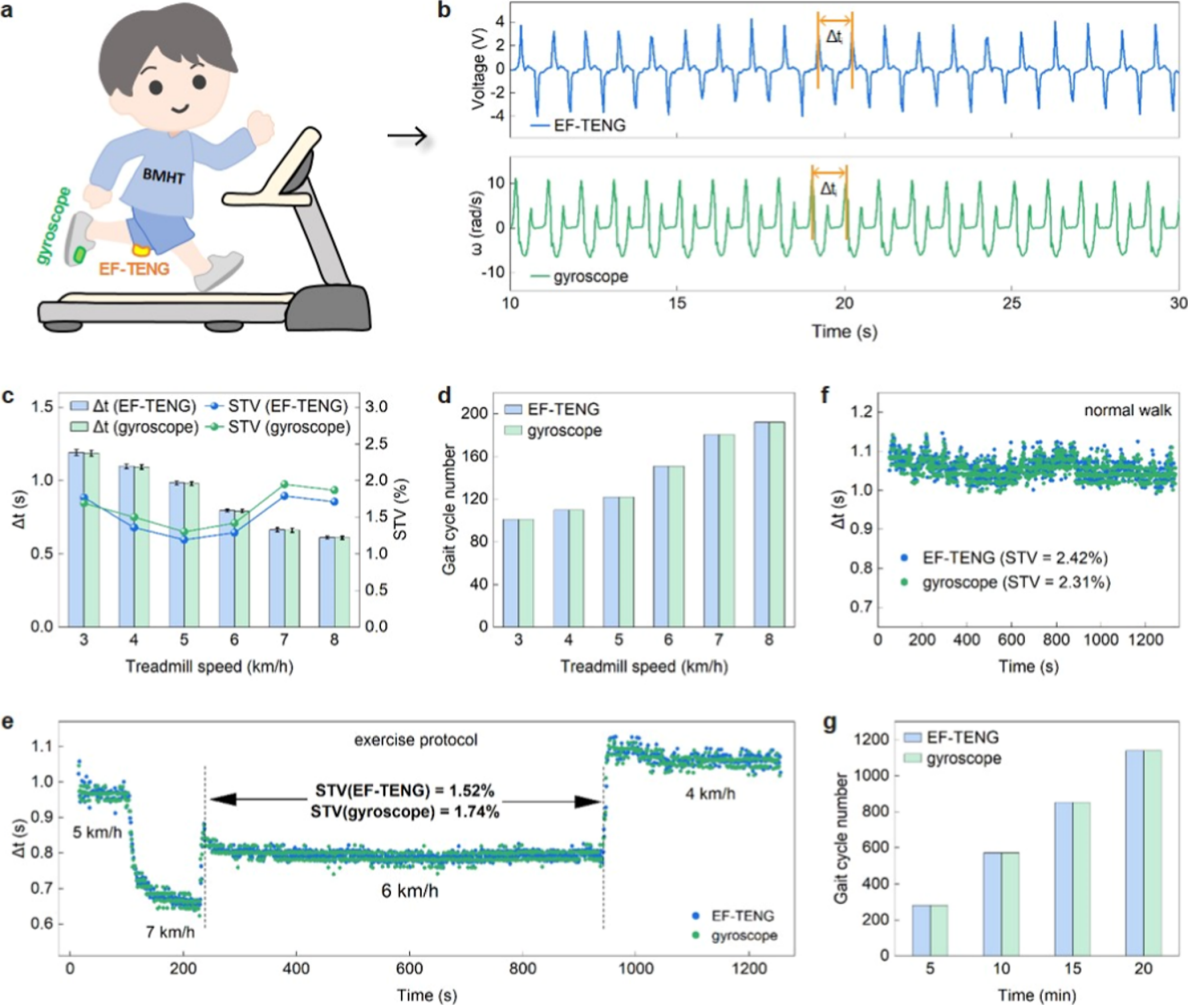
STV analysis with integrated triboelectric textiles via displacement current. (a) Schematic diagram of a runner wearing a smart garment with EF-TENG integrated near the knee and a gyroscope attached to the shoe. The responses with both methods, featuring the EF-TENG and gyroscope, are compared for motion analysis. (b) Responses from the EF-TENG and gyroscope during normal walking (where Δ*t*_*i*_ is the stride time indicated by the peak-to-peak interval); (c,d) comparison of runner’s average stride time (Δ*t*), stride time variability (STV), and gait cycle number at different treadmill speeds. Error bars indicate average and standard deviations of 200 readings. (e) Comparison of runner’s Δ*t* and STV obtained by continuously tracking motion during an exercise protocol. (f,g) Comparison of Δ*t*, STV, and gait cycle number during a prolonged walk of 20 min.

The physiological parameters of an adult runner were further monitored during a 20 min exercise protocol and a 20 min normal walking period. The EF-TENG reliably tracked motion over the entire duration and showed similar results to the gyroscope. The Δ*t* obtained with both methods showed close agreement, and the STV measured over a long duration with the smart garment and gyroscope were 1.52% and 1.74% (Figure 2e), respectively, indicating the runner’s healthy condition.^34,35^ During normal walking, the measured Δ*t* (a difference of 4 ms) and STV (a difference of 0.11%) with both methods were also similar (Figure 2f), followed by the same gait cycle number calculated every 5 min (Figure 2g). This demonstrates the potential of EF-TENG integrated garments and tReader to perform continuous physiological monitoring in real-life settings.

Despite our advances in EF-TENG integration and smart garment-based continuous physiological monitoring, hamstringing the convenience of our sensing system is the need for connectors to interact with the reader. To eliminate these connectors and further advance the ubiquitous use and transformative potential of EF-TENG-integrated garments, we adapted a fully textile wireless signal transfer approach utilizing solely textile components. By simply rearranging the textile wire connectors into a sewn-in inductor, we implemented a wireless sensing system with a boosted performance (Figure 3a). Specifically, a sewn-in conductive coil (∼3.25 μH, Figure S7) replaces the connectors, sending signals wirelessly to a reader inductor coil (∼3.35 μH, Figure S8) conveniently added to the existing tReader that transmits this information to a smartphone. The resistance of the textile connectors and the inductor remains low (1–2 Ω), ensuring efficient signal transmission and long-term durability while maintaining functionality even after washing, all with negligible added bulk to the garment.^36^

**Figure 3:**
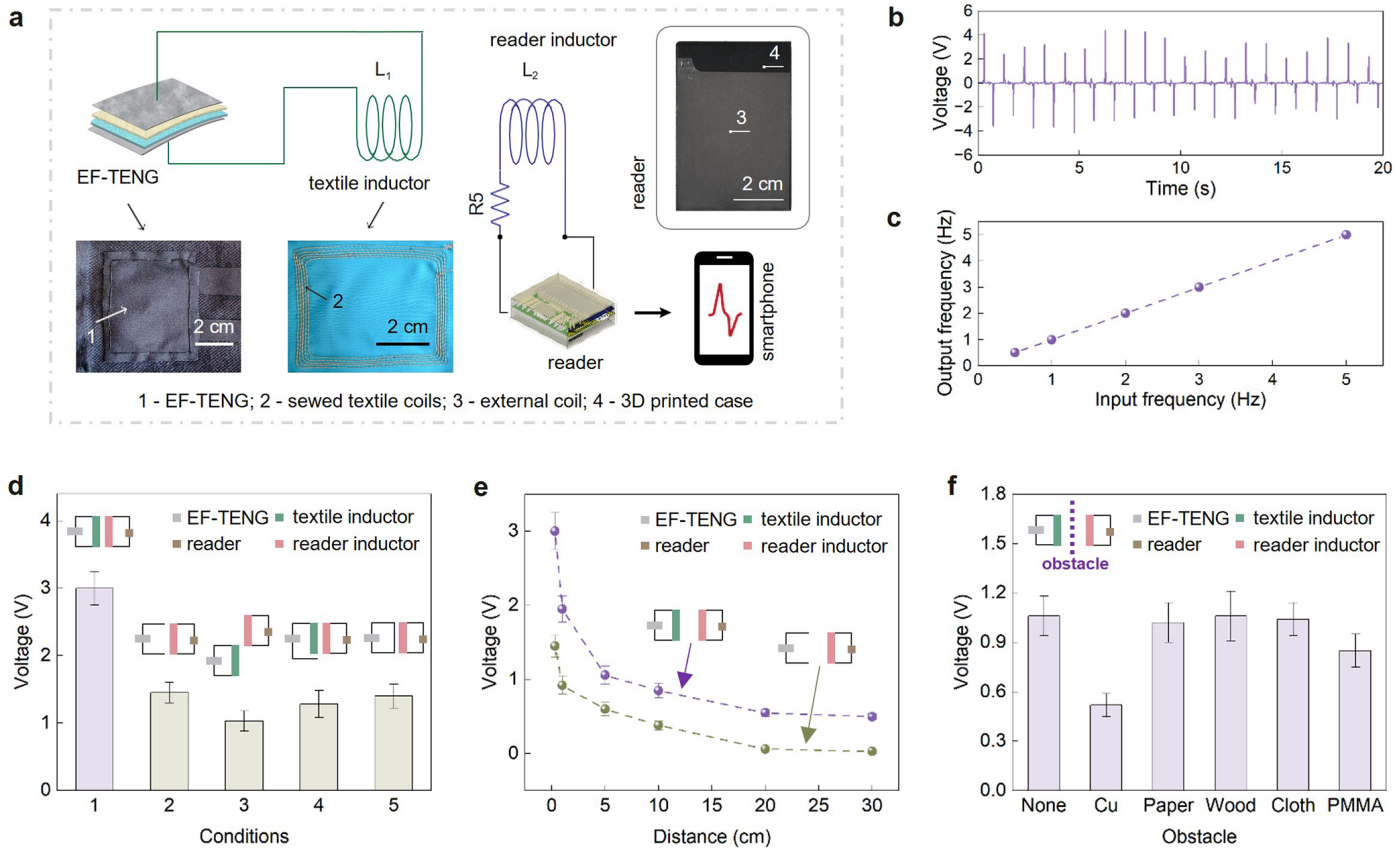
Wireless system via displacement current and the impact of inductor on wireless output signals. (a) Schematic of the wireless system incorporating inductors, where the textile inductor connected to EF-TENG wirelessly transfers signals to an external coil linked to the tReader that transmits this information to a smartphone app. (b) Voltage signal generated via normal working and received via displacement current. (c) Frequency of the output voltage signal from EF-TENG with respect to the frequency of the loading with Instron (pressure). (d) Comparison of responses with the wireless system under different conditions. (e) Influence of the separation distance between the transmitting and receiving inductors on the received voltage signal. (f) Influence of an obstacle placed midway between the transmitting and receiving inductors (separated by 5 cm) on the received voltage signal.

The corresponding mechanism can be explained as follows: the EF-TENG generates surface charges due to the contact and separation of its textile layers in response to the wearer’s body movements. The electrostatic field typically drives a conduction current through an external circuit when connected to a load. However, the internal circuit is primarily governed by the displacement current. Under no-load conditions, the term ∂*P*/∂t in the below equation, shown by Wang et al.^28^ to play a crucial role in displacement current, drives a dynamic electric field that enables wireless signal generation and transmission.

2 $$\frac{\partial D}{\partial t}=\varepsilon_{o}\frac{\partial E}{\partial t}+\frac{\partial P}{\partial t}$$

where *E* and *D* are the electric field and electric displacement field, respectively, and ε_o_ is the permittivity of free space. ∂*P*/∂t, arising from triboelectrically generated charges, contributes to the electrical signal emitted by the EF-TENG. The change in displacement current triggered by the EF-TENG’s mechanical action leads to time-varying electric fields, which can contribute to the generation of waves, carrying energy and enabling wireless signal transmission.

The open circuit voltage *V*_oc_(*t*) of the EF-TENG is given by^37^

3 $$V_{oc}\left(t\right)=\frac{\sigma x\left(t\right)}{\varepsilon_{o}}$$

where σ is the surface charge density and *x*(*t*) is the distance between triboelectric layers. The capacitance of the TENG varies as

4 $$C_{TENG}\left(t\right)=\frac{\varepsilon_{o}S}{d_{o}+x\left(t\right)}$$

where *S* is the electrode area and *d*_o_ is the effective thickness of EF-TENG. When the EF-TENG is connected to an inductor *L* and circuit resistance as *R*, Kirchhoff’s voltage law gives

5 $$V_{TENG}\left(t\right)=L\frac{di\left(t\right)}{dt}+Ri\left(t\right)$$

or equivalently

6 $$V_{oc}\left(t\right)-\frac{Q\left(t\right)}{C_{TENG}\left(t\right)}=L\frac{d^{2}Q\left(t\right)}{{dt}^{2}}+R\frac{dQ\left(t\right)}{dt}$$

Further,

7 $$\frac{dQ\left(t\right)}{dt}=C_{TENG}(t)\left[\frac{dV_{oc}\left(t\right)}{dt}+\frac{Q\left(t\right)}{C_{TENG}^{2}\left(t\right)}\frac{dC_{TENG}\left(t\right)}{dt}-L\frac{d^{3}Q\left(t\right)}{{dt}^{3}}-R\frac{d^{2}Q\left(t\right)}{{dt}^{2}}\right]$$

Equations 6 and 7 indicate that the presence of the inductor modifies charge redistribution by introducing a second-order differential term. The rapid signal variations in TENG pulses ensure that the inductor influences the circuit current. Specifically, the inductor slows the rate of charge transfer in the EF-TENG circuit, allowing more electric field (or displacement current) to build up during the contact and separation processes (in eq 2). While the inductor does not directly generate displacement current, it influences the system by providing more time for charge accumulation and variation, resulting in a stronger displacement current for the same mechanical motion. This increased displacement current enhances energy transfer from the TENG to the external circuit. When near a receiver coil, this increased displacement current $\frac{\partial D}{\partial t}$ induces a stronger response, improving coupling and enhancing the detected signal. Additionally, the orientation of the inductors further shapes the dynamic electric field, facilitating a better energy transfer.

To test the feasibility of wireless signal transfer with this system, we monitored an adult athlete’s motion during normal walking by integrating EF-TENG into the sock (detailed explanation can be found later.) As shown in Figure 3b and Video S2, the wireless system delivered a distinct voltage peak characteristic of the EF-TENG during normal walking. Notably, a nontriboelectric textile in place of EF-TENG did not deliver any such distinct peaks (Figure S9). This implies the possibility of wirelessly transmitting the signal solely with textile components and power generated with EF-TENG to the physically isolated receiving inductor of the reader. Furthermore, we also confirmed the system response by placing the textile and inductor coils facing each other and applying pressure to the EF-TENG (using an Instron). The wireless system as well produced alternating voltage spikes consistent with applied frequency upon load contact and release (Figure 3c), proving its ability to monitor slow-to-medium-paced body movements.

To elucidate the potential impact of the designed textile inductor on the circuit, we investigated various conditions, including scenarios with or without inductors, open or closed circuits, and different arrangements. With open-circuited textile connectors, the tReader wirelessly recorded a characteristic TENG signal similar to a wired approach at every pressing cycle of EF-TENG (Figure 2b). While open-circuited textile connectors allow signal transmission by TENG, tests reveal a 2-fold increase in signal strength with the textile inductor present (Figure 3d, conditions 1 and 2). This suggests that the presence of an inductor and the conduction current through it contribute significantly to an efficient wireless signal transfer. Misaligning the receiver coil from the transmitter’s plane (Figure 3d, condition 3) demonstrably weakens the received signal strength, highlighting the critical role of precise coil configuration. Also, an open-circuit configuration with the textile inductor present (Figure 3d, condition 4) and no conduction current flowing yields a weaker signal. Notably, also simply short-circuiting the textile connectors to allow direct conduction current (Figure 3d, condition 5) fails to achieve a comparable signal strength. The control experiments likely highlight the significance of the momentary switching of the textile inductor by the EF-TENG’s mechanical action. The nature of the inductor to oppose sudden change in current and the reorientation of charges may have likely influenced the transmitted fields, leading to an improved wireless signal transfer to the receiver coil. In addition, the separation distance and presence of obstacles between inductors affected the output voltage. The voltage received by the reader decreased as the distance between the inductors increased, reflecting the weakening of the propagating fields with distance (Figure 3e). To study the effect of different obstacles, we placed the receiver inductor 5 cm apart and introduced an object halfway through. Copper caused the lowest output, likely due to its strong attenuation of electromagnetic signals, among others (Figure 3f).

Environmental factors, such as temperature and humidity, can influence both EF-TENG performance and wireless transmission efficiency. As discussed in our previous work,^33^ EF-TENG output remains stable across a broad temperature range (−18–60 °C), demonstrating strong thermal resilience. However, increasing humidity from 20% to 50% RH and further to 74% RH led to a gradual decline in output performance, primarily due to charge dissipation. Similar trends were observed in wireless transmission performance, where temperature fluctuations had minimal impact, while higher humidity levels resulted in reduced transmission efficiency (Figure S10). Despite this decline, the system consistently captured clear single-peak signals, confirming its robustness and feasibility for operation under varying environmental conditions. These findings highlight the reliability of the EF-TENG-based wireless sensing system for practical applications across operating environmental conditions.

To continuously monitor similar motion metrics as previously mentioned, the EF-TENG was sewed around the sock’s anterior transverse arch. This explored its integration at a different location and facilitated the capture of sufficient walking pressure for wireless signal transfer. The textile inductor, positioned on the sock between the calf and ankle and connected to the EF-TENG with conductive threads, aligns with the reader’s inductor when placed in the adjacent stitched pocket (Figure 4a). Moreover, we placed a gyroscope on the foot for comparison when monitoring an adult athlete’s motion during normal walking. With each step, as illustrated in Figure 4b, the wireless system delivered a clear voltage peak, whose position confirmed the alignment of the signal from the gyroscope, indicating the accuracy of using our EF-TENG as the motion tracker and its great potential in STV analysis.

**Figure 4:**
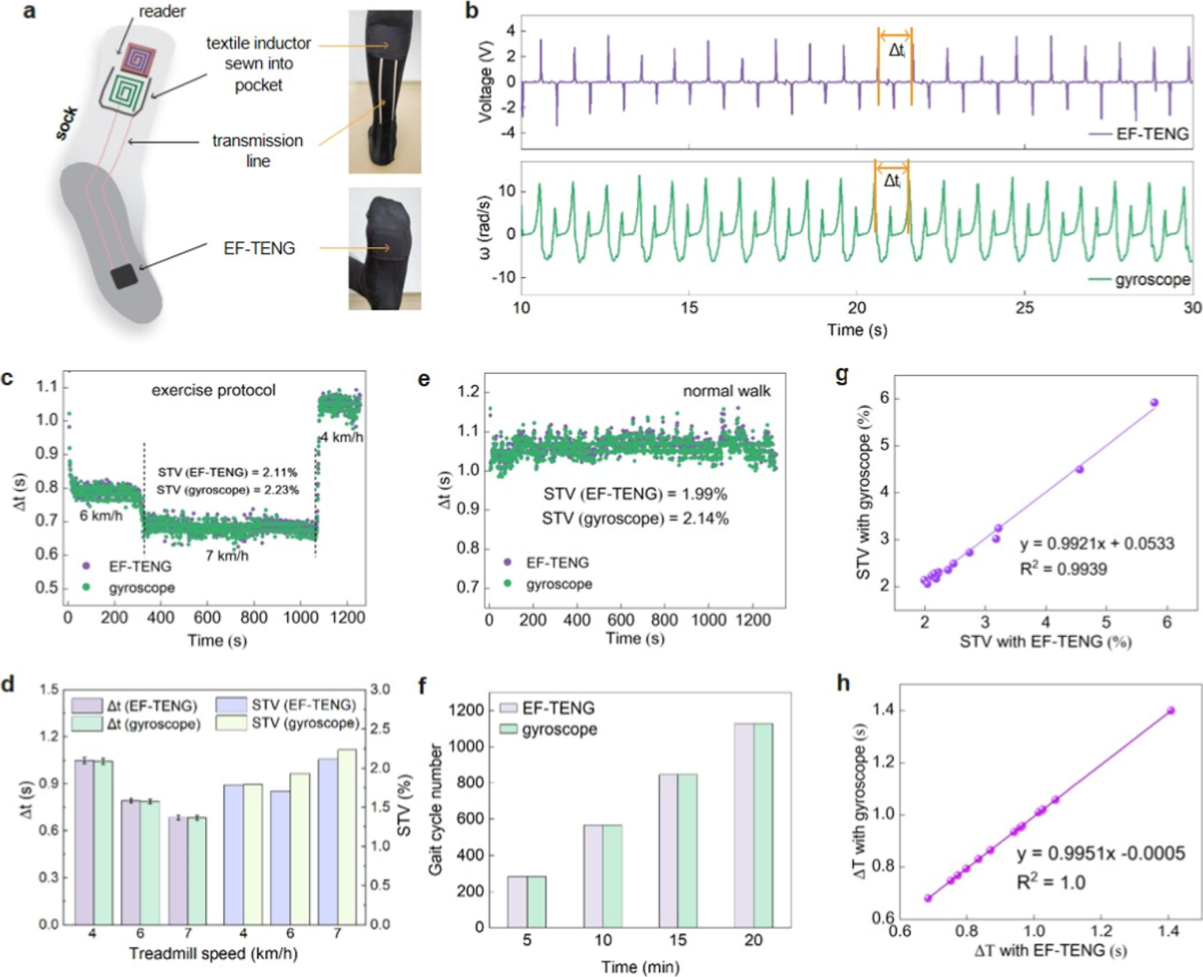
Wireless STV analysis with integrated triboelectric textiles via displacement current. (a) Schematic and photos illustrating the placements of EF-TENG, conductive textile transmission lines, and the textile inductor on the sock. The external reader coil aligns closely with the textile inductor when the reader sits in the stitched pocket. (b) Responses with EF-TENG and the gyroscope during a normal walk (where Δ*t*_*i*_ is the stride time indicated by the peak-to-peak interval). (c,d) Comparison of Δ*t* and STV obtained with an inductive wireless system and gyroscope during an exercise protocol. (e) Comparison of Δ*t* and STV during a 20 min walk. (f) Comparison of the gait cycle number during a 20 min walk. (g,h) Comparison of readings from the inductive wireless system and gyroscope, covering a broad range of STV and Δ*t* values across diverse participants.

Based on the above observations, we chose to place the inductors in proximity (separation of 2–3 mm) for continuous monitoring studies to ensure better signal quality and monitored an adult athlete’s motion during exercise and normal walking periods. The STV (2.11%) measured with the wireless system during exercise closely matched the readings with the gyroscope (2.23%) (Figure 4c,d). Similarly, for a normal walk, the system’s readings for Δ*t* (1.058 s), STV (1.99%), and the gait cycle number at 5 min intervals were comparable to those of the gyroscope (Δ*t* = 1.064 s, STV = 2.14%) (Figure 4e,f). Overall, the EF-TENG performed well under different conditions, delivering comparable results to the gyroscope. These results validate the feasibility and accuracy of our wireless system for continuous motion monitoring, contributing to a truly all-textile, comfortable smart garment experience.

To enhance the robustness and broader applicability of our findings, we conducted gait analysis on a diverse group of participants, ensuring variability in age, gender, and disability status (a subset of samples is shown in Figure S11). The inductive wireless system consistently demonstrated reliable performance across different conditions, with Δ*t* and STV values closely aligned with gyroscope measurements. This consistency highlights the system’s ability to accurately capture gait characteristics across individuals with varying biomechanical profiles. Additionally, validation of the EF-TENG wireless measurements through linear regression analysis revealed a strong correlation with gyroscope readings, yielding an *R* ^2^ value of ∼0.99 for STV and 1.0 for Δ*t* (Figure 4g,h). These results underscore the robustness and reliability of our approach, confirming its suitability for diverse real-world applications including continuous gait monitoring and movement analysis across various populations.

Beyond the lower-body gait analysis, we explored the applicability of our wireless TENG-based sensing system for upper-body movement tracking and performance monitoring. Specifically, we assessed JTV during repetitive elbow flexion-extension movements under both unloaded and loaded conditions (3–4 wt % of body weight), using gyroscope data for validation. Participants performed 20 continuous movement cycles per block, with a 30 s rest period between blocks, allowing for a controlled evaluation of neuromuscular adaptation and fatigue effects (Figure 5a–c). JTV was calculated as the coefficient of variation (CV) of intermovement time intervals, using TENG-generated voltage peaks as movement markers. Additionally, the mean movement duration (Δ*T*) was analyzed to assess the pacing changes across blocks. Results showed a progressive increase in both Δ*T* and JTV, indicating movement slowing and greater timing variability over time (a subset of samples is shown in Figure 5d–g). This trend was more pronounced under the loaded condition, particularly in participants P2 and P5, suggesting that external resistance exacerbates movement inconsistency. These findings indicate that movement consistency declines with repeated task execution, likely due to fatigue, neuromuscular adaptation, or shifts in motor control strategies. Our results demonstrate the potential of TENG-based sensors for real-time movement variability tracking with applications in rehabilitation (e.g., monitoring motor control during neurological recovery), sports training (e.g., optimizing movement consistency under fatigue), and fatigue detection for injury prevention. The strong correlation between TENG and gyroscope data, validated through linear regression (*R* ^2^ = ∼0.99 for JTV and 1.0 for Δ*T*; Figure 5h,i), further supports TENG-based sensing as a reliable biomechanical tracking tool.

**Figure 5:**
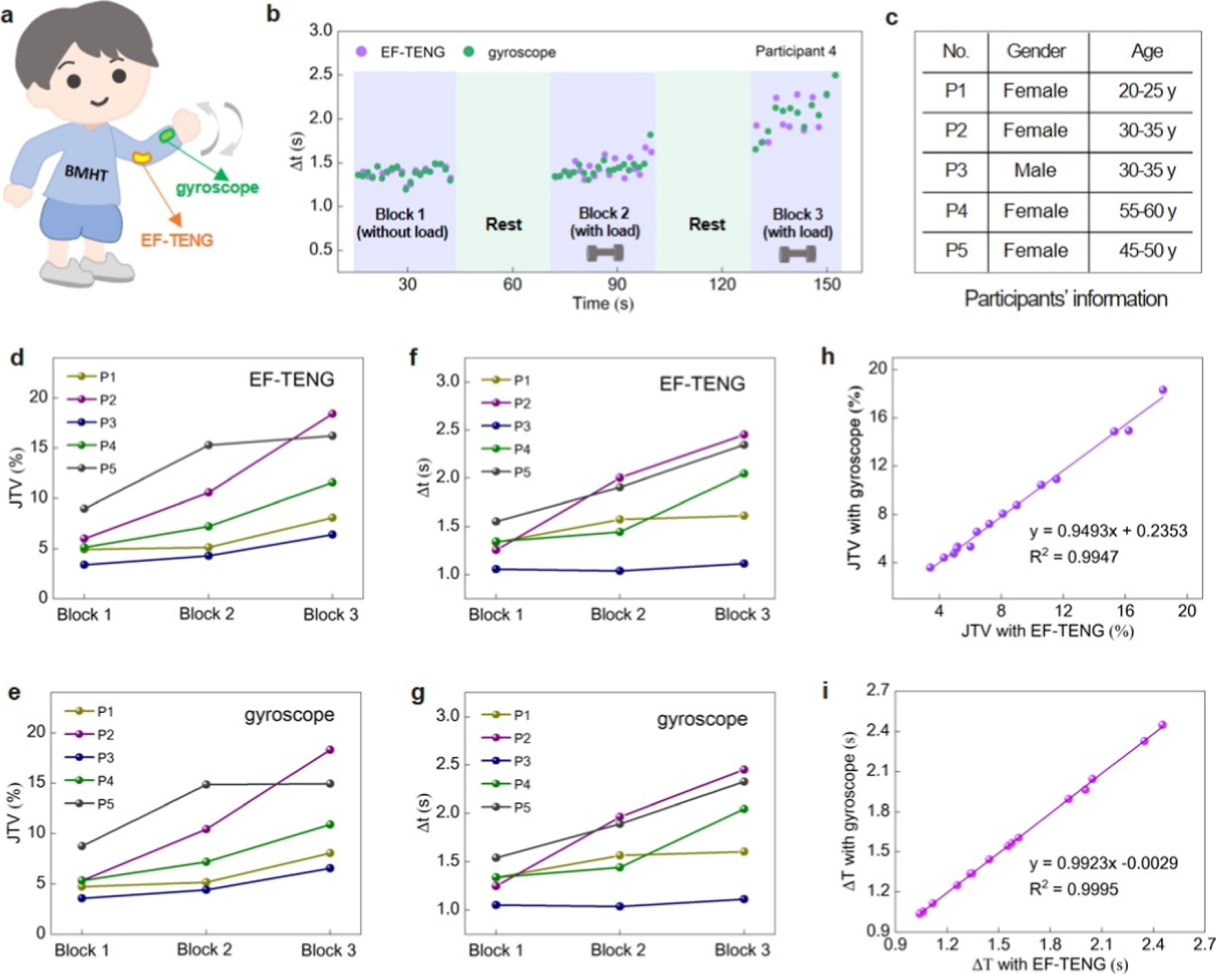
TENG-based sensing for upper-body movement tracking and performance monitoring. (a) Schematic diagram of a runner wearing a smart garment with EF-TENG integrated near the elbow and a gyroscope attached near the wrist. (b) Typical variation in movement duration across blocks during repetitive elbow flexion-extension movements under unloaded and loaded conditions (3–4 wt % of body weight). (c) Participants’ information in terms of gender and age. (d,e) JTV analysis highlighting increased variability over time, with a more pronounced effect under loaded conditions. (f,g) Δ*T* across blocks, showing a progressive increase in movement time. (h,i) Strong correlation between TENG-based sensing and gyroscope data, confirming the reliability of TENG for biomechanical tracking.

## 3 Conclusion

This work introduces a key advancement in textile wearables by achieving unobtrusive gait sensing and STV analysis and upper-body movement tracking through the seamless fusion of a full-textile triboelectric garment and a wireless communication architecture. The microgap-based EF-TENG integrated into the garment enabled detection of subtle movements at different body locations, opening doors for diverse applications in healthcare and beyond. While our EF-TENG wireless system shows great promise, several challenges must still be addressed for large-scale commercialization. These include the cost and complexity of specialized manufacturing equipment, the need for precise control of electrospinning parameters to ensure uniform fiber fabrication, and concerns about the fabrication efficiency for commercial production.

The wireless communication strategy harnessing displacement currents enabled wireless signal transmission using fully textile components, requiring no silicon components, external power sources, or physical connections with the reader. The presence of a textile inductor further enhanced the strength of the transmitted signal, and the EF-TENG and inductor embedded within the garment remained functional over extended use (over 10 months of storage). This ensures comfort and unobstructed mobility, further enhanced by the wireless interaction with the reader. The successful demonstration of this untethered triboelectric textile wireless system for continuous activity monitoring over extended periods lays the groundwork for a deeper understanding of human biomechanics and its application in various domains, from healthcare and sports science to human–computer interaction and remote patient monitoring.

## 4 Methods

### 4.1 Materials and Components

All chemicals, such as polyvinyl chloride (PVC), polycaprolactam (nylon), dimethylformamide (DMF), tetrahydrofuran (THF), and formic acid, were purchased from Sigma-Aldrich and used as received unless otherwise stated. All electronic components were purchased from DigiKey unless otherwise stated.

### 4.2 Synthesis of Electrospun Nanofibers

10 wt % amount of PVC powder was dissolved in a mixed solvent composed of 50% DMF and 50% THF. The obtained solution was placed into a syringe and then transformed into PVC nanofibers using an electrospinning machine (NS Plus, Inovenso) operated at 15 kV, with a 15 cm distance between the needle and collector and a flow rate of 0.5 mL/h. Similarly, 25 wt % of nylon pellets were dissolved in formic acid and used as the electrospinning solution to produce nylon nanofibers. The electrospinning parameters for nylon included an applied voltage of 25 kV, a 20 cm distance between the needle and collector, and a flow rate of 0.15 mL/h. Both the fabricated PVC and nylon nanofibers were collected on a conductive textile (Ag textile) with a spray glue pretreatment for subsequent use.

### 4.3 Fabrication and Integration of EF-TENGs

The EF-TENG was fabricated by overlaying and stitching a PVC/Ag textile and nylon/Ag textile together, with the PVC and nylon sides serving as the contact interface. To seamlessly integrate it into clothing, the fabricated EF-TENG was sewed directly onto specific areas of the garment, such as the elbow, knee, elbow pit, and knee pit and on the sock in this study. Notably, the size of the PVC/Ag textile was intentionally designed to be smaller than that of the nylon/Ag textile to prevent the connection of two conductive textiles during sewing and testing. Representative samples illustrating the integration of EF-TENG into clothing are shown in Figure 1d,e.

### 4.4 Fabrication of the Textile Inductor

The textile inductor was manufactured by sewing conductive thread in a rectangular loop (5.5 × 4.5 cm, 5 turns with a 1 mm gap between turns, Figure 3a) on a fabric using a sewing machine (NV2600, Innov-is). The resulting textile inductor was then stitched into a pocket and connected to the EF-TENGs via conductive textile transmission lines (Figure 4a).

### 4.5 Characterization and Instrumentation

The surface morphologies of the nanofibers were studied by a scanning electron microscope (GeminiSEM 450, ZEISS). The force source for pressure and velocity studies was provided by an electrodynamic test instrument (ElectroPuls E3000, Instron). The device output performance tests were conducted using an oscilloscope (MDO34, Tektronix) with a high-voltage probe (PR1050D, RIGOL) and a low-noise current preamplifier (SR570, Stanford Research Systems). It is noted that the high-resolution oscilloscope mode was employed in this work, averaging samples for improved signal quality and reduced noise, albeit sacrificing peak amplitude compared to peak-detection mode. This trade-off is crucial for accurate sensing in slow-to-medium movements (Figure S12 demonstrates much higher peaks with peak-detection mode than with high-resolution mode).

## Supplementary Material







## Data Availability

The data that support the findings of this study are available from the corresponding author on reasonable request.

## Notes

Impacts of fabric electrodes, climbing motion, nontriboelectric textiles, environmental factors, and detecting mode on the device output or wireless transmission; SEM images of triboelectric nanofibers; frequency consistence between EF-TENG and Instron; device reproducibility and long-term stability; impedance characterization of textile and commercial inductors; and STV and Δ*t* analysis across a diverse population (PDF)
Motion tracking with a triboelectric smart textile (MP4)
Wireless motion tracking with a triboelectric smart garment (MP4)
