# Supplementary material for: Wireless Motion Variability Analysis with Integrated Triboelectric Textiles via Displacement Current
Source: ACS Nano. 2025 May 23;19(22):20539–49. doi: 10.1021/acsnano.4c18766 (PMC12164512; doi:10.1021/acsnano.4c18766)
Supplement: Supplementary file 1 [file nn4c18766_si_001.pdf]

SUPPORTING INFORMATION

Wireless Motion Variability Analysis with Integrated Triboelectric Textiles via Displacement Current

Yinghong Wu[1,2,#,*], *Sunil Kumar Sailapu*[1,#,*], *Chiara Spasiano*[1,3], *Carlo Menon*[1,*]

[1]Biomedical and Mobile Health Technology Group, Department of Health Sciences and Technology, ETH Zürich, Lengghalde 5, Zürich 8008, Switzerland.

[2]National Engineering Research Center of Green Recycling for Strategic Metal Resources, Institute of Process Engineering, Chinese Academy of Sciences, Beijing 100190, China

[3]School of Industrial and Information Engineering, Politecnico di Milano, Milan 20133, Italy.

[#]These authors contributed equally.

*Email: yhwu@ipe.ac.cn, sunil.sailapu@hest.ethz.ch, carlo.menon@hest.ethz.ch

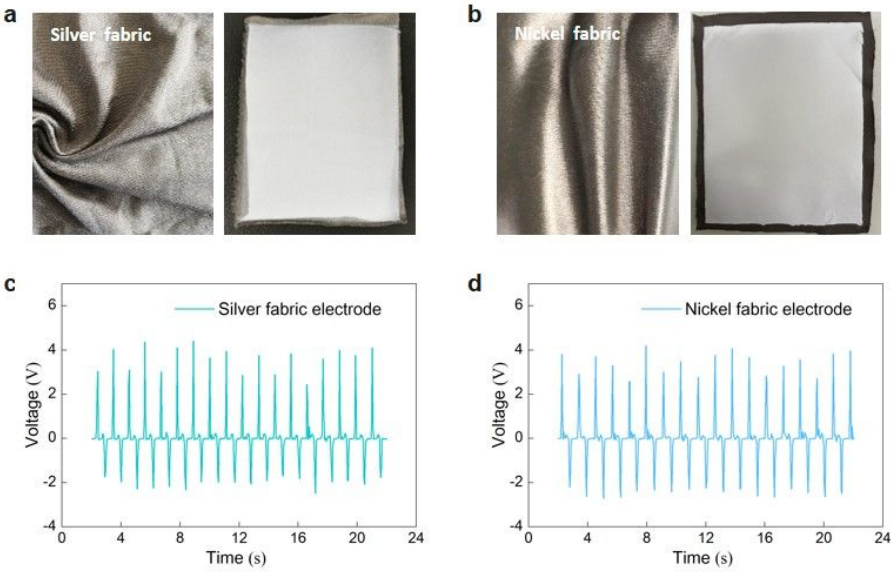

Figure S1. Effect of conductive fabric electrodes on the device output.

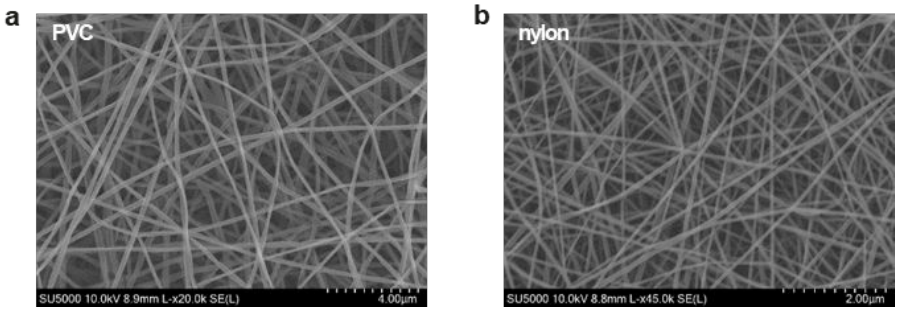

Figure S2. SEM images of PVC and nylon nanofibers.

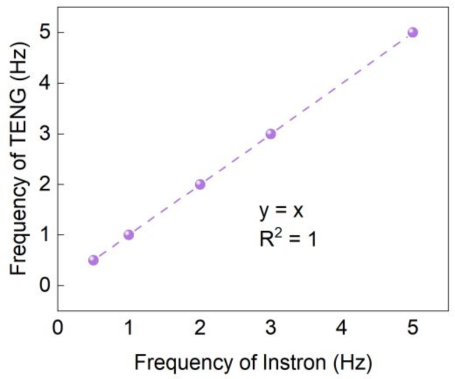

Figure S3. Frequency of the output voltage signal from EF-TENG with respect to the frequency of the loading with Instron (pressure).

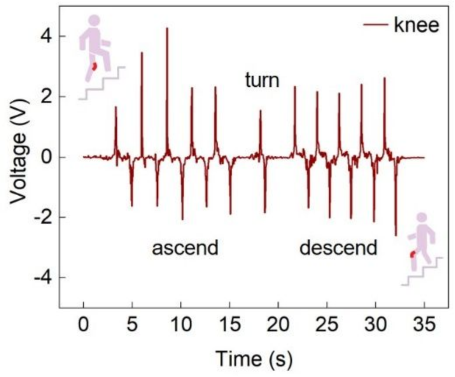

Figure S4. Voltage generated during motion on an inclined plane, i.e., ascending and descending the stairs.

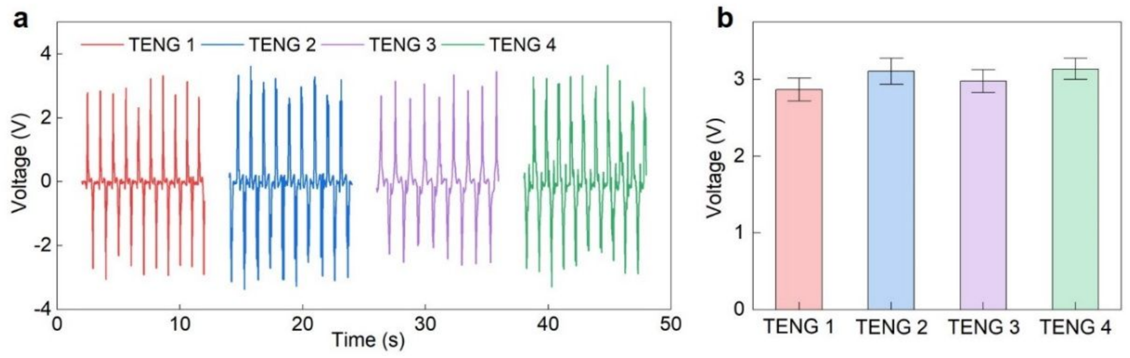

Figure S5. a) Voltage signals and their b) average peak values from four separate EF-TENG integrated garments during normal walking.

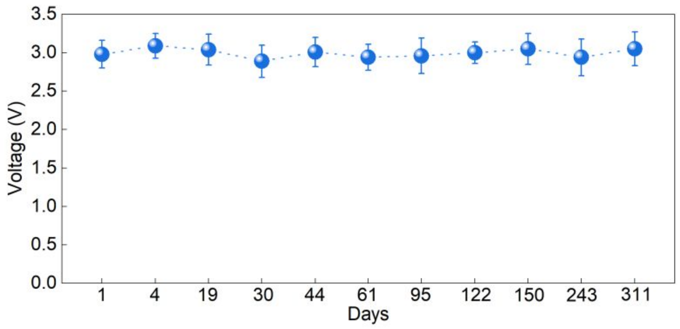

Figure S6. Average peak voltage of the EF-TENG integrated garment over a 10-month period.

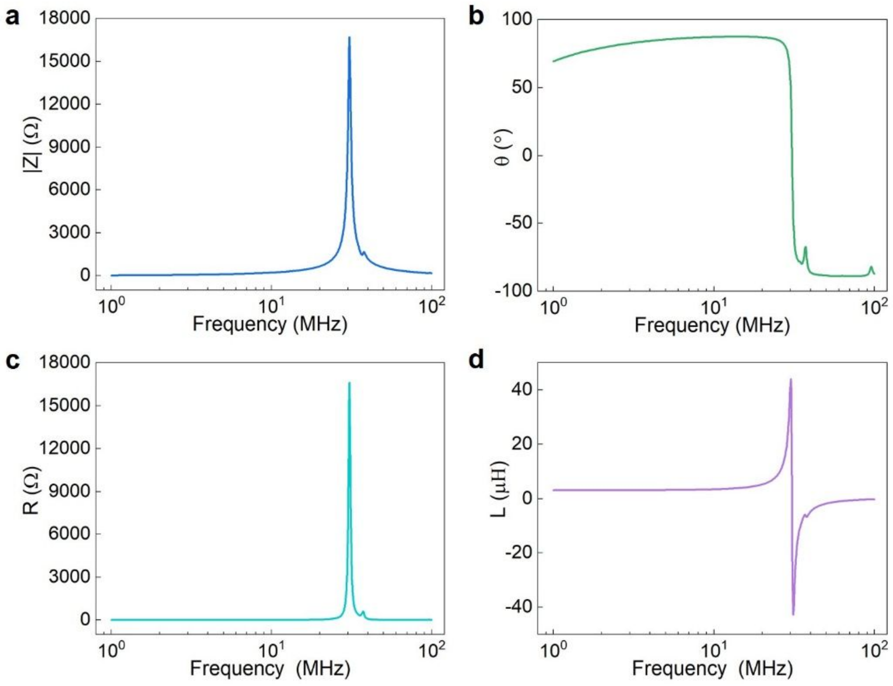

Figure S7. Impedance characterization of the textile inductor. a,b) Magnitude and phase of the textile inductor's impedance. c,d) Magnitude of the resistive and inductive component of the textile inductor.

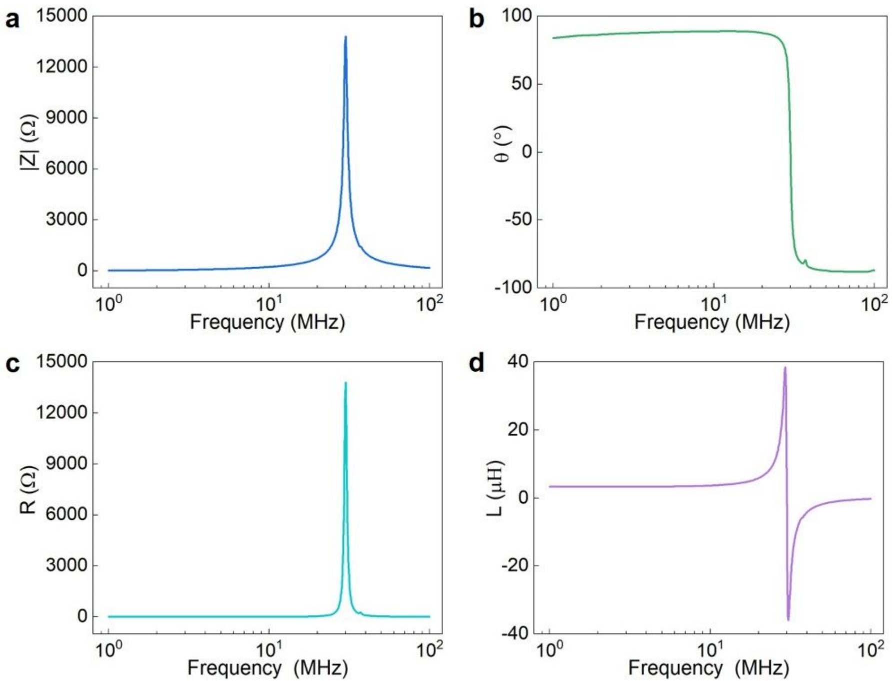

Figure S8. Impedance characterization of the commercial inductor of the tReader. a,b) Magnitude and phase of the tReader inductor's impedance. c,d) Magnitude of the resistive and inductive component of the tReader inductor.

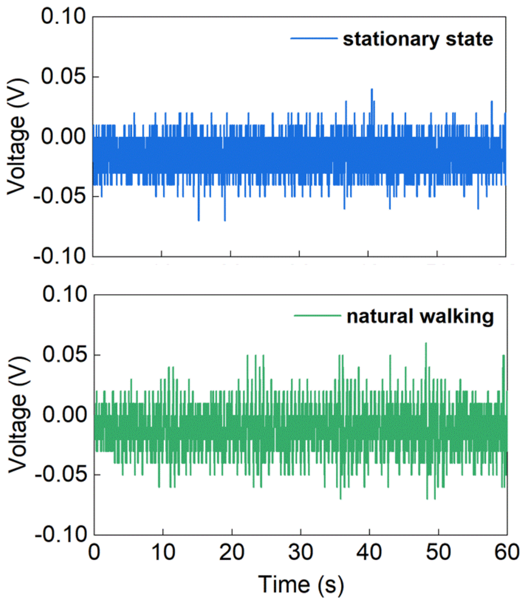

Figure S9. Effect of non-triboelectric textile on the voltage signal during a) stationary state (baseline), and b) natural walking.

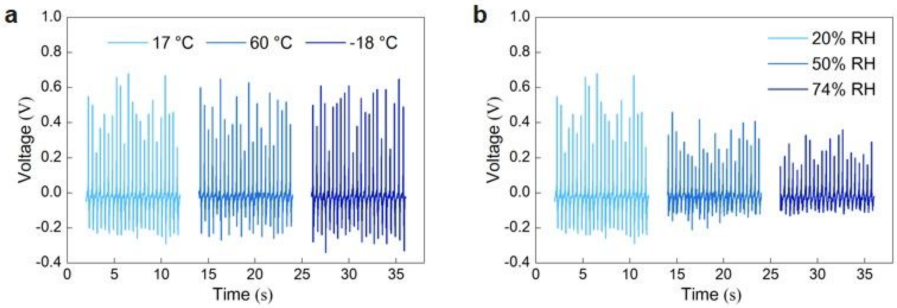

Figure S10. Effects of environmental factors on wireless transmission. a) Voltage responses at different temperatures (17 ℃: in the lab; 60 ℃: in the oven; -18 ℃: in the freezer.). b) Voltage responses at different humidity levels (20% RH: in the lab; 50% RH and 74% RH: with humidifier in a closed chamber). Energy source: finger tapping at ~45-50 kPa.

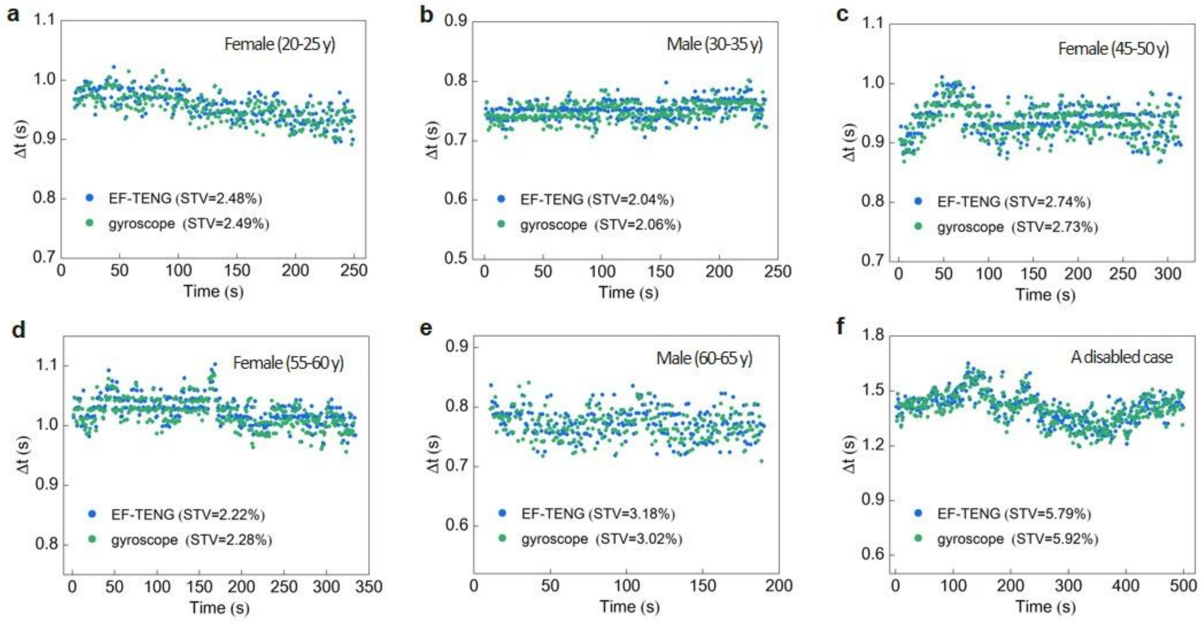

Figure S11. STV and Δt analysis across a diverse population. a-f) Comparison of Δt and STV measured using the inductive wireless system and gyroscope during normal walking across different participants (readings > 200).

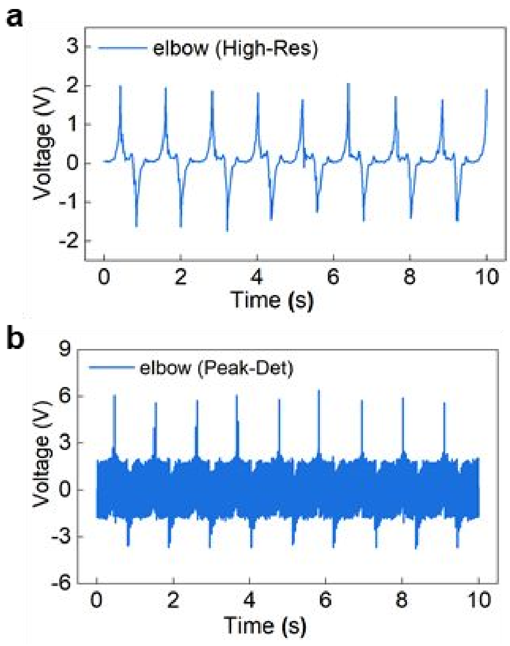

Figure S12. Voltage signals from EF-TENG measured with oscilloscope in a) high-resolution and b) peak detection mode.
